# Prediction of oral diseases in care dependent older people

**DOI:** 10.1186/s12903-024-05261-y

**Published:** 2025-01-11

**Authors:** Christina Andersen, Astrid Pernille Jespersen, Kim Ekstrand, Esben Boeskov Øzhayat

**Affiliations:** 1 Department of Odontology, Faculty of Health and Medical Sciences, University of Copenhagen, Nørre Allé 20, Copenhagen, 2200 Denmark; 2 The Saxo Institute, Copenhagen Centre for Health Research in Humanities, University of Copenhagen, Karen Blixens Plads 8, Copenhagen, 2300 Denmark

**Keywords:** Oral health, Dental care for aged, Frail older adults, Health services for the aged

## Abstract

**Background:**

A large number of older people depend on others for help with their daily personal care, including oral health care. Nursing home and elder-care staff often face challenges identifying older people, who are exposed to or at an increased risk of oral diseases. Thus, the aim of this study was to identify risk factors that non-dental care staff can use to identify older people at risk of oral diseases and poor oral hygiene.

**Methods:**

In this cross-sectional study, the oral health and risk factors for poor oral health were determined for 217 care dependent older people living in two nursing homes and a rehabilitation centre or receiving home care in two Danish municipalities. The outcome variables for oral disease i.e. caries, periodontitis, and gingivitis, and oral hygiene, i.e. plaque and calculus, were assessed using standardised oral examinations. Risk factors for oral diseases and poor oral hygiene were assessed based on a questionnaire concerning (1) background information, (2) health status, (3) social support, (4) oral health behaviours, and (5) self-reported oral health. The associations between oral health and risk factors were investigated using logistic regression analyses.

**Results:**

In general, older people with different living arrangements had quite similarly distributed risk factors. The oral examinations showed that 54.5% had oral diseases, and 14.8% had poor oral hygiene. Not seeing a dentist regularly was significantly associated with having oral diseases (Odds Ratio, 2.87; CI, 1.53–5.39) and poor oral hygiene (OR, 4.50; CI, 1.83–11.05). A significant association was found between the presence of an oral disease and adversely affected quality of life (OR, 2.65; CI, 1.42–4.95), especially due to challenges eating (OR, 3.76; CI, 1.64–8.60) and/or smiling and showing teeth (OR, 3.64; CI, 1.27–10.42). A significant association was also found between poor oral hygiene and taking psychotropic drugs (OR, 2.61; CI, 1.08–6.30).

**Conclusion:**

Questions regarding the use of the dental care system and oral health problems could be used by nondental care staff in conversations with older people to determine their risk of oral diseases and poor oral hygiene.

## Background

A growing number of older people aged 65 or older are living with comorbidity and a high medical intake in Denmark, as in most of the world [1]. Further, natural teeth are frequently retained in these frail older people [2], posing challenges for good oral hygiene and leading to a disturbingly high prevalence of oral diseases [2]. Oral diseases can lead to pain, tooth loss, discomfort, and impaired eating ability, which can result in weight loss, malnutrition, social isolation, and a reduced quality of life [2]. Oral diseases can further aggravate several general diseases, such as diabetes and cardiovascular disease [2], and poor oral hygiene is a frequent reason for aspiration pneumonia and hospitalisation in frail older people [3].

In Denmark, approximately 40,000 older people live in nursing homes, and approximately 128,000 older people receive home care [4]. Of these, approximately 13,000 receives personal care, 58,000 practical assistance and 57,000 both [4]. These older people depend on others for help with their daily personal care, including oral hygiene, and can be considered frail in this regard. Furthermore, their vulnerable situation often results in a lack of attachment to the dental care system [5], making their next-of-kin or care staff best placed to discover and act on oral health problems. Unfortunately, nursing home and home-care staff are often extremely busy and lack knowledge about oral conditions and diseases [6]. Thus, they have difficulty identifying older people who are exposed to or at an increased risk of oral diseases [6].

Several studies have shown that educational interventions aimed at care staff may have some short-term effects, but there is limited evidence of their long-term effectiveness [7]. Furthermore, many of these studies have focused only on nursing home residents or hospitalised people [7], limiting the generalisability of studies by not including community-dwelling older people. This has resulted in missed preventative opportunities.

To identify frail older people in need of oral care, oral assessment screening tools, such as the Revised Oral Assessment Guide (ROAG) [8] and the Oral Health Assessment Tool (OHAT) [9], have been developed for use by nondental staff [8, 9]. Although these screening tools are considered sensitive [9], they are also quite comprehensive and require both staff time and training. The limited time and resources of older people’s carers can make such tools difficult to implement, and their existence has not decreased the prevalence of oral diseases in older people. Simpler methods of oral screening for frail older people are needed and should be based on knowledge of the relationship between poor oral health status and common factors that are easily accessible in the care of older people.

The aim of this study was to identify risk factors that can help nondental care staff identify older people at increased risk of oral diseases and poor oral hygiene. Identifying such factors will give care staff a better basis for recognising oral health issues in their daily work and acting upon them. This could support the prevention and treatment of oral diseases and improve the general health and quality of life of older people.

## Methods

### Setting

This cross-sectional study is part of the Lifelong Oral Health research project, which aims to develop guidelines for improving oral health in frail older people. The study was conducted in two Danish municipalities (Frederiksberg and Greve) located in the capital region of Denmark. Frederiksberg municipality has approximately 105,000 citizens and is in central Copenhagen—the largest city and capital of Denmark. Greve is a suburban municipality south of Copenhagen with approximately 51,000 citizens. Approximately 17% of the population in Frederiksberg and approximately 21% in Greve are 65 years or older [10], and both municipalities have increasing numbers of older people. These municipalities were selected because of their previous experience with health improvement projects. In each of the municipalities one ward in a nursing home and one home care unit, taking care of community dwelling citizens, was included. In Frederiksberg municipality, an inpatient rehabilitation centre responsible for treating older people with declining functions or in need of rehabilitation following hospitalisation was also included. In total, five care units were involved in the study.

### Participants and recruitment

All older people registered with the involved nursing homes, rehabilitation centre or receiving home care from the involved home care units were considered for participation. The care staff determined the older people’s suitability for participation, excluding any they considered too cognitively deficient or unhealthy, and noting the reasons for non-participation. Information was collected on the older people’s age, sex, and level of dependence. The same information was collected for the older people, who declined to participate which emerged from the information material.

In the nursing homes and the rehabilitation centre, the care staff recruited the participants by informing older people and/or their relatives about the project and asking them to participate. For community-dwelling older people, the home-care staff handed out an information letter and requested permission for the researcher to call the citizens to ask about participation. Informed consent was collected from all participants or from their legal guardians.

We were aware that the participants might be classed as vulnerable people, and to minimise adverse effects, we conducted the examinations in the older people’s own homes, offering a family member or home-care staff to be present to give support. The participants were told that they could withdraw from the examinations at any time. We do not believe that the examinations negatively affected the participants. If oral diseases were found, the care staff were informed accordingly.

**Fig. 1:**
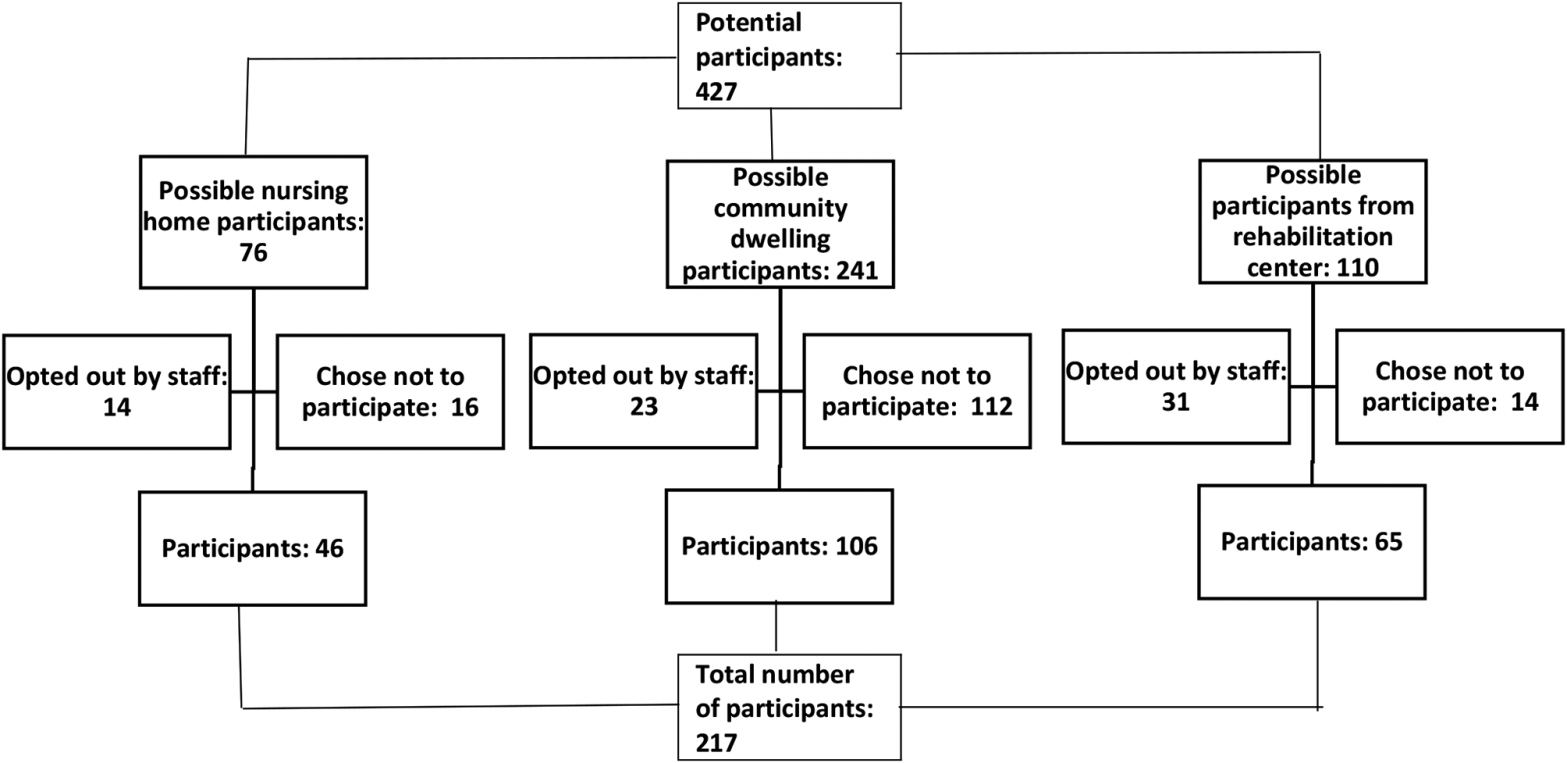
Flowchart of participant selection

A total of 427 potential participants between 53 and 98 years of age were identified, 217 of whom were included in the study. The questionnaire was answered by 215 participants and 207 had the oral examination. This imply that some of the variables were not available for all participants. Of the 217 participants, 46 were nursing home residents, 65 were registered with the rehabilitation centre, and 106 were community dwellers (Fig. 1). Since the participants from the five units had comparably distributed risk factors and oral health statuses, to achieve adequate data, they were analysed as a single group.

## Data collection and study variables

### Oral status

Oral health status was the outcome variable and encompassed two variables for oral disease and oral hygiene. These variables were assessed using standardised oral examinations. The first author (C. A.) performed all the examinations following a self-calibration process, which involved examining the first 20 older people twice to ensure agreement and consistency across examinations. In the case of non-agreement, a third examination was performed. For the examinations, standardised dental examination equipment was used, including a mouth mirror, a dental probe, and a periodontal probe. A headlamp and cotton rolls were used to improve the visual inspections. However, it was not possible to do radiological examinations in the participants´ own homes.

The variable for oral disease was based on the observation of dental caries, periodontal disease, and/or bleeding from the gums. Caries was recorded when the participant needed operative caries therapy (i.e. fillings) because a tooth surface was clinically cavitated. This was in accordance with the International Caries Detection and Assessment System (ICDAS) [11] dichotomized into no caries (score 0–2: healthy surface or lesions in the enamel) and caries (score 3–6: carious loss of tissue or dentin caries).Periodontal disease was determined according to the Community Periodontal Index of Treatment Needs (CPITN) [12] for six sites in the mouth (upper right, upper middle, upper left, lower right, lower middle, and lower left), and each site was assigned a score of 0–4. Periodontal disease was recorded when the participant had at least one site with a score of 3 or 4, meaning a loss of bone attachment. The condition of the gums (gingivitis) was determined using the Löe and Silness Gingival Index (GI-index) [13]: six index teeth (teeth 16, 12, 24, 32, 36, and 44) were examined with a probe, and each tooth surface was assigned a score of 0–3. A score was calculated for each tooth, and then an overall average score of 0–3 was given for each participant. The condition of the gums was recorded as good (*≤* 1.0), fair (1.1–2.0), or poor (*≥* 2.1). For analytical purposes, the gum condition was dichotomised as good/fair or poor. An oral disease was considered present if a participant had a minimum of one caries lesion, one site with periodontal disease, or if the condition of the gums was poor.

Oral hygiene was assessed using the Oral Hygiene Index Simplified (OHI-S) [14] to determine dental plaque and calculus on six index teeth (teeth 16, 11, 26, 31, 36, and 46). How large an area of each tooth was covered with plaque and calculus (none, 1/3, 1/3–2/3, > 2/3) was recorded, and each tooth was given a score of 0, 1, 2, or 3. Based on this scoring, an oral hygiene index was calculated as an average score of 0–6 across the six index teeth. For analytical purposes, oral hygiene was dichotomised as good/fair and poor, with poor being an OHI-S score *≥* 3,1 [14].

### Risk factors

The risk factors for oral diseases and poor oral hygiene were determined using a questionnaire. The questionnaires were done as interviews by the first author to ensure their correct understanding of the questions and to minimise missing data. Risk factors were categorised according to (1) background information, (2) health status, (3) social support, (4) oral health behaviours, and (5) self-reported oral health.

Risk factors related to background information included sex, age (< 80 years or *≥* 80 years of age), living conditions (nursing home, rehabilitation centre, or community dwelling), and level of dependence (receiving help with personal care from care staff or not). Personal care could include for example, help with bathing, dressing and in some cases tooth brushing.

Risk factors relating to health status were determined using questions drawn from the Copenhagen Aging and Midlife Biobank study [15] and the Danish Health Examination Survey [16]. The questions dealt with general health issues and the use of medications known to affect oral health. The health issues recorded were neurodegenerative disorders (e.g., Parkinson’s disease or dementia) and long-term mental illness (longer than 6 months). Registered medications were inhalers for asthma/chronic obstructive pulmonary disease, psychotropic drugs, and polypharmacy (five or more different) drugs. The care staff provided information on health issues and medication if the participants could not give this information themselves.

Social support was assessed by asking the participants about the degree to which they received practical help or support from relatives, i.e., spouse, children, other family members, or friends. The answer categories were ‘always’, ‘often’, ‘sometimes’, ‘rarely’, ‘never’, and ‘no relatives or friends’ [15]. For analytical purposes, the variable was dichotomised as high support (always, often, and sometimes) and low support (rarely, never, and no relatives or friends).

Oral health behaviours were recorded for smoking (yes/no), sugary diet, tooth brushing, and regularity of seeing a dentist. A sugary diet was based on the reported intake of cake, juice, soda, marmalade, chocolate, candy, and sugar in coffee and tea. The response categories were as follows: several times per day, once daily, several times per week, once per week, several times per month, or never. For analytical purposes, the answers were dichotomised as a daily sugary diet (several times per day and daily) or not a daily sugary diet (several times per week, once per week, several times per month, and never). Tooth brushing was categorised as more than once per day, once per day, a couple of times per week, a couple of times per month, or never. For analytical purposes, this was dichotomised as daily or less than daily tooth brushing. The researchers further recorded whether the participants were receiving assistance with tooth brushing from care staff. Whether the participants visited dentists was determined by asking questions drawn from the Copenhagen Aging and Midlife Biobank study [15] regarding how often each participant had seen a dentist within the last five years. The answer categories were a minimum of once per year, 3–4 visits, 1–2 visits, or no visits within the last five years. The variable was dichotomised as seeing a dentist regularly (a minimum of once a year within the last five years) or irregularly (less than yearly visits to a dentist, i.e., 3–4 visits, 1–2 visits, or no visits within the last five years).

Risk factors related to self-reported oral health were determined by asking questions regarding oral health-related quality of life (OHRQoL), toothache, and dry mouth. OHRQoL was assessed using the Oral Impact on Daily Performance (OIDP) questionnaire [17] to determine how often the following factors proved problematic: eating and enjoying food, speaking clearly and pronouncing words, cleaning the teeth, sleeping/relaxing without oral pain or discomfort, smiling/showing the teeth without embarrassment, mood, and enjoying social interaction with others. For each factor, the frequency of experiencing a problem was rated as never, less than once per month, 1–2 times per month, 1–2 times per week, or daily. Adversely affected quality of life was defined as experiencing any of the mentioned problems at any frequency. The factors smiling/showing teeth, ability to eat, and social interaction were also included as individual risk factors, since these factors generally have the highest incidence of negatively affecting quality of life according to the OIDP [18]. Toothache was assessed by asking about pain in the teeth and mouth within the last six months (yes/no). Dry mouth was evaluated using questions from the Clinical Oral Dryness Score questionnaire [19] regarding experiences of dry mouth in general. The answers were divided into yes (all the time or most of the time) and no (never, rarely, and sometimes).

### Data analysis

Statistical analyses were performed using IBM^®^ SPSS^®^ software version 29.0.1.0 (171). The significance level was set at *p* < 0.05. Logistic regression analyses were used to examine the associations between the presence of an oral disease, poor oral hygiene, and risk factors. For models with oral disease as an outcome, the individual risk factors were included in individual models, adjusting for frequency of seeing a dentist, tooth brushing, and sugary diet, since these factors have the greatest impact on oral diseases. For the models with oral hygiene as an outcome, the individual risk factors were also included in individual models, adjusting for daily tooth brushing only, as this was considered to have the greatest impact on oral hygiene.

## Results

### Participants

The distribution of risk factors (Table 1) showed that most participants were female (59%), 80 years old or older (62.5%), and received personal care (72.8%). In general, the participants had received help and support from their relatives or friends (88.4%), and more than half reported seeing a dentist regularly (57.7%). Nearly all reported brushing their teeth at least once a day (93.5%), and only a few received help with brushing their teeth (8.8%). Most of the participants reported consuming sugar daily (78.6%), and some were smokers (13.5%). In general, the living arrangements were comparable across the groups in terms of the distribution of risk factors. Nursing home residents, however, took more medications, had more frequent mental illness and neurodegenerative disorders, and were more often smokers than the two other groups.

**Table 1:** Distribution of risk factors. Distribution of the participants’ risk factors according to living arrangements and in total

	Nursing home residents (46)	Community dwelling (106)	Rehabilitation centre (65)	Total
				-217
**Sex**				
Female	27 (58.7%)	62 (58.5%)	39 (60.0%)	128 (59.0%)
Male	19 (41.3%)	44 (41.5%)	26 (40.0%)	89 (41.0%)
**Age group**				
< 80 years	14 (31.1%)	47 (44.3%)	20 (30.8%)	81 (37.5%)
*≥* 80 years	31 (68.9%)	59 (55.7%)	45 (69.2%)	135 (62.5%)
**Level of dependence**				
Not personal care	10 (21.7%)	34 (32.1%)	15 (23.1%)	59 (27.2%)
Personal care	36 (78.3%)	72 (67.9%)	50 (76.9%)	158 (72.8%)
**Inhalation medicine**				
No	43 (95.6%)	84 (80.0%)	54 (83.1%)	181 (84.2%)
Yes	2 (4.4%)	21 (20.0%)	11 (16.9%)	34 (15.8%)
**Psychotropic drugs**				
No	27 (58.7%)	93 (88.6%)	51 (78.5%)	171 (79.2%)
Yes	19 (41.3%)	12 (11.4%)	14 (21.5%)	45 (20.8%)
**Polypharmacy**				
No	25 (54.3%)	62 (58.5%)	43 (66.2%)	130 (59.9%)
Yes	21 (45.7%)	44 (41.5%)	22 (33.8%)	87 (40.1%)
**Mental illness**				
No	30 (66.7%)	94 (90.4%)	55 (84.6%)	179 (83.6%)
Yes	15 (33.3%)	10 (9.6%)	10 (15.4%)	35 (16.4%)
**Neurodegenerative disorder**				
No	33 (73.3%)	93 (88.6%)	57 (87.7%)	183 (85.1%)
Yes	12 (26.7%)	12 (11.4%)	8 (12.3%)	32 (14.9%)
**Support from next of kin**				
Low	8 (17.8%)	10 (9.5%)	7 (10.8%)	25 (11.6%)
High	37 (82.2%)	95 (90.5%)	58 (89.2%)	190 (88.4%)
**Dental care habits, seeing a dentist**				
Regularly	25 (55.6%)	60 (57.1%)	39 (60.0%)	124 (57.7%)
Irregularly	20 (44.4%)	45 (42.9%)	26 (40.0%)	91 (42.3%)
**Tooth brushing**				
Daily	44 (95.6%)	97 (92.4%)	61 (93.8%)	202 (93.5%)
Less than daily	2 (4.4%)	8 (7.6%)	4 (6.2%)	14 (6.5%)
**Assisted tooth brushing**				
Yes	8 (17.8%)	5 (4.8%)	6 (9.2%)	19 (8.8%)
No	37 (82.2%)	100 (85.2%)	59 (90.8%)	196 (91.2%)
**Sugary diet**				
Not daily	9 (20.0%)	19 (18.1%)	18 (27.7%)	46 (21.4%)
Daily	36 (80.0%)	86 (81.9%)	47 (72.3%)	169 (78.6%)
**Smoking**				
No	35 (77.8%)	91 (86.7%)	60 (92.3%)	186 (86.5%)
Yes	10 (22.2%)	14 (13.3%)	5 (7.7%)	29 (13.5%)

Of the non-participants, 40.4% of those who were not asked to participate and 57.7% of those who declined to participate were female, and 57.4% of those not asked and 50.1% of those declining to participate were 80 years old or older. Most of the participants in both groups (82.8%) received personal care. The main reasons for not being asked to participate were cognitive or other health challenges, and the main reasons for declining participation were general unwillingness to participate in research and a lack of the necessary strength and energy to do so.

### Oral status

Table 2 shows that 25.6% of the participants had a minimum of one caries lesion that needed treatment. Furthermore, 40.2% had periodontal disease, and 7.9% had poor gum condition and bleeding gums. In total, 54.5% had an oral disease, and 14.8% had poor oral hygiene.

**Table 2:** Description of the participants´ oral health statuses. Description of oral status according to living arrangements and total

	Nursing home residents (46)	Community dwelling (106)	Rehabilitation centre (65)	Total
				-217
**Oral hygiene**				
Good	11 (28.2%)	27 (28.7%)	15 (26.8%)	53 (28.1%)
Fair	22 (56.4%)	55 (58.5%)	31 (55.4%)	108 (57.1%)
Poor	6 (15.4%)	12 (12.8%)	10 (17.8%)	28 (14.8%)
**Condition of gums**				
Good	17 (43.6%)	33 (34.7%)	21 (37.5%)	71 (37.4%)
Fair	21 (53.8%)	54 (56.9%)	29 (51.8%)	104 (54.7%)
Poor	1 (2.6%)	8 (8.4%)	6 (10.7%)	15 (7.9%)
**Periodontal disease**				
Yes	17 (43.6%)	36 (37.9%)	23 (41.8%)	76 (40.2%)
No	22 (56.4%)	59 (62.1%)	32 (58.2%)	113 (59.8%)
**Dental caries**				
Yes	12 (27.3%)	24 (23.1%)	17 (28.8%)	53 (25.6%)
No	32 (72.7%)	80 (76.9%)	42 (71.2%)	154 (74.4%)
**Toothache**				
Yes	9 (20.0%)	19 (18.1%)	6 (9.2%)	34 (15.8%)
No	36 (80.0%)	86 (81.9%)	59 (90.8%)	181 (84.2%)
**Oral disease**				
Yes	24 (61.5%)	46 (48.4%)	33 (60.0%)	103 (54.5%)
No	15 (38.5%)	49 (51.6%)	22 (40.0%)	86 (45.5%)

### Associations between oral disease and risk factors

Only a few of the risk factors examined were significantly associated with oral disease. Table 3 shows that none of the background factors were statistically significantly associated with oral disease in the adjusted analyses. Being male was associated with an increased risk of having an oral disease in the unadjusted analysis (OR, 1.94; CI, 1.06–3.54).

**Table 3:** Associations between oral disease and risk factors. Results of regression analyses examining the associations between oral disease as an outcome and the different risk factors as explanatory variables (significance level 0.05)

Risk factors	Association with oral disease, crude	Association with oral disease, adjusted*
	OR 95% CI *P*	OR 95% CI *P*
**Background information**		
**Sex**		
Female	Ref.	Ref.
Male	1.94 1.06–3.54 0.03	1.72 0.92–3.22 0.09
**Age group**		
< 80	Ref.	Ref.
*≥* 80	1.47 0.81–2.65 0.20	1.77 0.94–3.34 0.08
**Living arrangement**		
Not nursing home residents	Ref.	Ref.
Nursing home residents	1.44 0.70–2.96 0.32	1.41 0.67–2.99 0.37
**Level of dependence**		
Help with personal care	Ref.	Ref.
No help with personal care	1.28 0.67–2.44 0.46	1.22 0.62–2.39 0.56
**Health status**		
**Inhalation medicine**		
No	Ref.	Ref.
Yes	0.53 0.24–1.19 0.13	0.50 0.22–1.16 0.11
**Psychotropic drugs**		
No	Ref.	Ref.
Yes	1.80 0.88–3.71 0.11	1.61 0.76–3.41 0.21
**Polypharmacy**		
No	Ref.	Ref.
Yes	1.13 0.63–2.02 0.68	1.08 0.59–1.98 0.81
**Mental illness**		
No	Ref.	Ref.
Yes	0.86 0.39–1.91 0.71	0.61 0.26–1.45 0.27
**Neurodegenerative disorders**		
No	Ref.	Ref.
Yes	1.01 0.47–2.20 0.97	1.00 0.45–2.25 0.99
**Social support**		
**Support from next of kin**		
High	Ref.	Ref.
Low	0.74 0.28–2.01 0.56	1.22 0.41–3.62 0.72
**Oral health behaviours**		
**Dental care habits, seeing a dentist**		
Regularly	Ref.	Ref.
Irregularly	2.98 1.60–5.55 < 0.001	2.87 1.53–5.39 <0.001
**Tooth brushing**		
Daily	Ref.	Ref.
Less than daily	2.33 0.60–9.06 0.22	1.64 0.40–6.82 0.49
**Assisted tooth brushing**		
No	Ref.	Ref.
Yes	0.70 0.24–2.16 0.53	0.71 0.22–2.28 0.56
**Sugary diet**		
Not daily	Ref.	Ref.
Daily	0.92 0.46–1.85 0.82	0.98 0.47–2.03 0.96
**Smoking**		
No	Ref.	Ref.
Yes	1.93 0.79–4.71 0.15	1.61 0.63–4.09 0.32
**Self-reported oral health**		
**Affected quality of life**		
No	Ref.	Ref.
Yes	3.11 1.70–5.71 <0.001	2.65 1.42–4.95 0.002
**Challenges eating**		
No	Ref.	Ref.
Yes	4.04 1.80–9.04 <0.001	3.76 1.64–8.60 0.002
**Challenges smiling and showing the teeth**		
No	Ref.	Ref.
Yes	4.92 1.79–13.55 0.002	3.64 1.27–10.42 0.02
**Challenges being social**		
No	Ref.	Ref.
Yes	3.06 0.62–15.13 0.17	2.07 0.40-10.82 0.39
**Dry mouth**		
No	Ref.	Ref.
Yes	2.06 0.85–5.01 0.11	2.39 0.95–5.97 0.06
**Toothache within the last 6 months**		
No	Ref.	Ref.
Yes	0.69 0.32–1.48 0.34	0.59 0.26–1.32 0.20

*Adjusted for dental care habits, daily tooth brushing and sugary diet

Regarding the risk factors related to health status and social support, not seeing a dentist regularly was significantly associated with oral disease after adjustment, with an OR of 2.87 (CI, 1.53–5.39).

Regarding the risk factors related to self-reported oral health, there was a significant association in the adjusted model between having an oral disease and an adversely affected quality of life (OR, 2.65; CI, 1.42–4.95). Having problems with eating and enjoying food and smiling/showing the teeth were also significantly associated with oral disease, with OR values of 3.76 (CI, 1.64–8.60) and 3.64 (CI, 1.27–10.42), respectively.

### Associations between oral hygiene and risk factors

In general, very few of the risk factors were significantly associated with poor oral hygiene (Table 4). It was found that not seeing a dentist regularly was significantly associated with poor oral hygiene (OR, 4.50; CI, 1.83–11.05) in the adjusted model. Furthermore, taking psychotropic drugs was significantly associated with poor oral hygiene (OR, 2.61; CI, 1.08–6.30). Smoking had a significant association with poor oral hygiene (OR, 2.76; CI, 1.03–7.44), although this became statistically insignificant after adjustment.

**Table 4:** Associations between oral hygiene and risk factors. Results of the regression analyses examining the association between poor oral hygiene as an outcome and the different risk factors as explanatory variables (significance level 0.05)

Risk factors	Association with oral hygiene, crude	Association with oral hygiene, adjusted*
	OR 95% CI *P*	OR 95% CI *P*
**Background information**		
**Age group**		
< 80	Ref.	Ref.
*≥* 80	0.77 0.34–1.74 0.53	0.79 0.34–1.85 0.59
**Sex**		
Female	Ref.	Ref.
Male	1.73 0.77–3.88 0.18	1.75 0.76–4.02 0.19
**Living arrangement**		
Not nursing home residents	Ref.	Ref.
Nursing home residents	1.06 0.40–2.82 0.91	1.20 0.44–3.24 0.72
**Level of dependence**		
No help with personal care	Ref.	Ref.
Help with personal care	1.38 0.52–3.63 0.52	1.38 0.52–3.67 0.52
**Health status**		
**Inhalation medicine**		
No	Ref.	Ref.
Yes	0.99 0.31–3.10 0.98	1.02 0.32–3.22 0.98
**Psychotropic drugs**		
No	Ref.	Ref.
Yes	2.47 1.03–5.91 0.04	2.61 1.08–6.30 0.03
**Polypharmacy**		
No	Ref.	Ref.
Yes	1.22 0.54–2.72 0.64	1.35 0.59–3.07 0.48
**Mental illness**		
No	Ref.	Ref.
Yes	2.18 0.83–5.76 0.12	2.03 0.76–5.46 0.16
**Neurodegenerative disorders**		
No	Ref.	Ref.
Yes	0.86 0.27–2.68 0.79	0.89 0.28–2.79 0.84
**Social support**		
**Support from next of kin**		
Low	Ref.	Ref.
High	0.39 0.13–1.20 0.10	0.44 0.14–1.41 0.17
**Oral health behaviours**		
**Dental care habits, seeing a dentist**		
Regularly	Ref.	Ref.
Irregularly	4.66 1.92–11.34 <0.001	4.50 1.83–11.05 0.001
**Tooth brushing**		
Daily	Ref.	Ref.
Less than daily	2.38 0.59–9.58 0.22	2.38 0.59–9.58 0.22
**Assisted tooth brushing**		
No	Ref.	Ref.
Yes	0.47 0.06–3.81 0.48	0.51 0.06–4.09 0.52
**Sugary diet**		
Not daily	Ref.	Ref.
Daily	2.49 0.71–8.73 0.15	2.87 0.79–10.46 0.11
**Smoking**		
No	Ref.	Ref.
Yes	2.76 1.03–7.44 0.04	2.62 0.96–7.14 0.06
**Self-reported oral health**		
**Affected quality of life**		
No	Ref.	Ref.
Yes	0.95 0.42–2.17 0.91	0.91 0.40–2.08 0.82
**Challenges eating**		
No	Ref.	Ref.
Yes	1.92 0.79–4.67 0.15	1.81 0.73–4.45 0.20
**Challenges smiling and showing the teeth**		
No	Ref.	Ref.
Yes	0.94 0.30–2.95 0.91	0.84 0.26–2.72 0.77
**Challenges being social**		
No	Ref.	Ref.
Yes	1.75 0.34–8.90 0.50	1.66 0.32–8.59 0.54
**Dry mouth**		
No	Ref.	Ref.
Yes	0.43 0.10–1.94 0.27	0.46 0.10–2.09 0.32
**Toothache within the last 6 months**		
No	Ref.	Ref.
Yes	0.57 0.16-2.00 0.38	0.53 0.15–1.91 0.33

*Adjusted for daily tooth brushing

## Discussion

In general, only a few of the risk factors used in this study had significant associations with oral diseases or poor oral hygiene. However, irregular visits to a dentist were an important risk factor for both poor oral hygiene and oral diseases. Moreover, reported oral problems and taking psychotropic drugs were important factors associated with oral diseases and poor oral hygiene, respectively.

The risk factors included in this study were expected or reported to be associated with poor oral health or poor oral hygiene. It was therefore somewhat surprising that only a few of the risk factors were significantly associated with oral health status, but they made sense.

Shimazaki et al. [20] found a similar association between dental consultations and oral health status, since participants with fewer dental visits had more dental caries and more deep periodontal pockets [20]. Treasure et al. [21] likewise found that people who consulted a dentist only when they had symptoms had a significantly higher risk of dental caries and unrestorable decay than those who visited dentists regularly.

Regarding oral hygiene, another study found a significant correlation between visiting a dentist regularly and having better oral hygiene practices [22]. Regarding self-reported oral problems as a risk factor for oral diseases, other studies have shown that older people with oral diseases such as ulcers, dental caries, periodontal disease, and bleeding from the gums, more frequently report poor OHRQoL and adversely affected daily performance [18, 23–26].

Several studies have revealed a high prevalence of oral diseases among people with psychiatric problems who take psychotropic drugs [27, 28] due to poor oral hygiene and the side effects of medication, especially dry mouth. In our study, we found an association between poor oral hygiene and taking psychotropic drugs, but we did not find a significant association between poor oral hygiene and mental illness, and the association with dry mouth was only borderline significant.

Unlike our study, other studies have found significant associations between oral health, smoking, and age [21, 29]. Konradsen et al. [29] discovered that smoking and age predicted a need for oral care in medical patients with acute conditions admitted to hospital. We included only care-dependent older people aged 65 or over. These were all ‘older’ people, so it was not surprising that age was not a significant factor. Regarding smoking, we found a borderline significant association between smoking and poor oral hygiene. The limited number of smokers among the participants might have led to an insignificant association.

Some studies have reported significant associations between being female and having oral diseases [18, 25], while another study found that men tended to have poorer oral hygiene and more oral diseases than women [30]. In our study, we found a significant association between being male and having an oral disease, but this association became statistically insignificant in the adjusted model, suggesting that other factors are more important than gender for oral health.

The nonsignificant associations between neurodegenerative disorders, oral disease, and poor oral hygiene in this study contrasts with another study indicating that declining cognitive functions are related to poor oral hygiene [31]. This difference is probably due to the use of different approaches for determining cognitive functions. In our study, we relied on a neurodegenerative disorder diagnosis, which may not be as sensitive as a specific cognitive evaluation. Furthermore, the nursing staff screened potential participants for cognitive ability prior to participation, which might have reduced the number of older people with cognitive impairments included in the study. This may have led to the underestimation of oral problems and made it difficult for us to obtain significant results, affecting the external validity of the study due to selection bias.

This study has other limitations. Since the examinations were performed in the participants´ private homes rather than in dental clinics, we were unable to conduct radiological examinations. This meant that not all oral diseases were necessarily diagnosed, and this probably led to the underestimation of oral diseases. In addition, in our study, we constructed a combined variable for oral disease that comprised caries, periodontitis, and poor gum condition (gingivitis); thus, we only considered the presence or absence of an oral disease and not the type of oral disease. Although this led to less detailed findings, regardless of the disease, citizens with oral diseases could still be identified and proper treatment initiated. Another limitation was the rather low number of participants. This resulted in small subgroups, making it difficult to identify significant differences. Furthermore, it limited the possibility of adjusting for all relevant factors in the logistic regression analyses, although the factors that were adjusted for were considered the most relevant. Also, recall bias may have been present, as much of the study relied on self-reported information, and due to some of the older people’s declining cognitive functions, they may not have remembered incidents correctly.

A strength of the study is that all the participants were seen by the same examiner, which ensured consistent data. Another strength was that the questionnaire was also completed using interviews, which ensured the participants’ correct understanding of the questions and minimised missing data. Many studies have been conducted in nursing homes only, whereas this study also included older people in a rehabilitation centre and community-dwelling older people, which resulted in a diverse and more representative sample. We found that older people with different living arrangements had comparably distributed risk factors and oral statuses, perhaps due to the participants being frail and care dependent, regardless of their living arrangements. Including community-dwelling older people facilitated a focus on prevention since initiatives to improve older people’s oral health would benefit from early detection of oral problems, preferably before they lead to deterioration in oral health.

### Implications for practitioners

The knowledge that self-reported oral problems and the irregular use of the dental care system are associated with poor oral health offer care staff a simple and practical way of identifying older people with oral health issues. It seems that simply asking citizens about these issues could result in better identification of older people with poor oral health. This knowledge could then be used to help decision-makers and managers in the elder-care field focus on oral health and timely prevention and treatment, with significant potential for early identification and outreach. Municipal home-care staff have contact with many older home-dwelling citizens who could benefit from improved oral care initiatives. This calls for prospective studies to test our findings and underpin such initiatives, since this study was cross-sectional and indicated associations rather than causality. If such initiatives identified more citizens with poor oral health, they could lead to fewer oral diseases and thus improve older people’s general health and quality of life.

## Conclusion

This study showed that asking questions about oral problems and dental visits can help care staff identify older people with oral health and oral hygiene issues. More studies are needed to test the potential of this knowledge as a screening method for identifying older people who need help with their oral care.

## Data Availability

The datasets used and/or analysed during the current study are available from the corresponding author upon reasonable request.
